# Enhancing efficiency and improving turnaround time: real-world impact of the Genius Digital Diagnostics System implementation

**DOI:** 10.1093/ajcp/aqag081

**Published:** 2026-07-21

**Authors:** Kristina Weatherhead, Chinh Nguyen, Terry Henry, Sarah Harrington, Yan Lemeshev

**Affiliations:** ProPath, A Sonic Healthcare Anatomic Pathology Practice, Dallas, TX, United States; ProPath, A Sonic Healthcare Anatomic Pathology Practice, Dallas, TX, United States; ProPath, A Sonic Healthcare Anatomic Pathology Practice, Dallas, TX, United States; Scientific Affairs, Hologic, Inc, Marlborough, MA, United States; ProPath, A Sonic Healthcare Anatomic Pathology Practice, Dallas, TX, United States

**Keywords:** digital cytology, digital pathology, artificial intelligence, cervical cancer, cytology, cytopathology, workflow, laboratory efficiency, Papanicolaou test, Genius Digital Diagnostics

## Abstract

**Objectives:**

We sought to assess the real-world impact of the Genius Digital Diagnostics System (Genius Dx [Hologic, Inc]) on workload, efficiency, and turnaround time in a high-volume cervical cytology laboratory.

**Methods:**

The laboratory information system was retrospectively queried for all Papanicolaou test cases performed between January 1, 2023, and March 31, 2025. A total of 512 177 cases (655 468 reviews) were included. Genius Dx was implemented on August 1, 2024, and cases were classified as preimplementation or postimplementation. Daily caseload, number of reviewers, number of cases per reviewer, and accession–to–sign-out times were compared using independent-samples *t* tests. Diagnostic distributions were compared using ꭓ^2^ tests.

**Results:**

Average daily cytologist (CT) reviews were similar before and after Genius Dx (747.8 vs 758.4 cases) but required fewer CTs per day (10.4 vs 8.1; *P* < .001), increasing cases per CT per day from 74.5 to 94.7 (*P* < .001). Pathologist reviews per day increased (106.0 vs 145.3; *P* < .001) with fewer pathologists (4.4 vs 3.4; *P* < .001). Average daily CT case workload increased by 53.5% after implementation. Accession–to–sign-out times decreased for all diagnoses (*P* < .001). A small but statistically significant shift in diagnostic proportions was observed, with a decrease in negative results and an increase in atypical squamous cells of uncertain significance or higher (ASC-US+) findings with negligible effect size.

**Conclusions:**

Implementation of Genius Dx substantially increased reviewer efficiency and reduced turnaround times without compromising diagnostic performance. The increased detection of ASC-US+ findings observed in this study are in line with results from the Genius Dx clinical trial and other published literature.

KEY POINTS

- Genius Dx increased CT and pathologist caseload per day, enabling the same overall volume with fewer reviewers.
- Accession–to–sign-out time fell across all major diagnostic categories, with turnaround time decreased most for high-grade (ASC-H+) cases.
- Diagnostic mix shifted slightly (fewer negative results, increased ASCUS+ findings), indicating improved disease detection without major category drift.

## INTRODUCTION

Screening with the Papanicolaou test has widely been credited with substantially reducing cervical cancer incidence and mortality by enabling the detection and treatment of precancerous lesions and early-stage cancers of the cervix.^1^^,^^2^ Although screening in the United States has continued to evolve with the inclusion of human papillomavirus testing, cytology screening remains integral to cervical cancer screening and triage.^3^ There has been substantial cytology innovation since the introduction of the Papanicolaou test in the 1950s, such as the introduction of liquid-based cytology and the automated imaging systems that have become a mainstay in cytology laboratories.^4‐6^ Despite these improvements, cytology laboratories continue to face major challenges.

Cytologist (CT) resources continue to be one of the main constraints cytology laboratories face. Workforce shortages,^7^^,^^8^ expanding CT roles and responsibilities,^9^ increased screening volumes, and short turnaround time targets all contribute to the need for solutions to this growing issue.

Recent developments in digital cytology and artificial intelligence offer promising solutions to address these limitations.^10^^,^^11^ The Genius Digital Diagnostics System (Genius Dx [Hologic, Inc]) was recently cleared by the US Food and Drug Administration^12^ to image ThinPrep Papanicolaou test slides (Hologic, Inc) and create a gallery of clinically relevant cells that CTs and pathologists can use to interpret cases. By enabling immediate digital access to cases and focused review of a gallery of clinically relevant cells , Genius Dx has the potential to streamline workflow, reduce screening time, and facilitate remote sign-out capabilities. The benefits of the Genius Dx may help mitigate the challenges today’s cytology laboratories face.

Several publications^13‐16^ have demonstrated that the Genius Dx is validated for gynecology cytology, demonstrating that the Genius Dx is accurate in the detection of cytologic abnormalities. Although review time is not the primary purpose of these studies, they have also reported that review times are decreased using Genius Dx compared with manual or ThinPrep Imaging System (Hologic, Inc) review. In addition, a recent workflow efficiency publication from our laboratory demonstrated the efficiency of the Genius Dx in reducing the hands-on time needed for glass slide movement as well as the time needed for screening and signing out cytology cases.^17^ This study, however, used a simulated workflow and as a result is limited in its real-world applicability.

The current study evaluated CT and pathologist workload and efficiency following the implementation of Genius Dx in a high-volume cytology laboratory using real-world preimplementation and postimplementation data obtained through a retrospective review of the laboratory information system (LIS). Performance was assessed by comparing the number of cases reviewed per day and the turnaround time of screening results before and after Genius Dx adoption, providing evidence for the system’s effect on laboratory throughput and workflow efficiency.

## METHODS

The LIS was queried to identify all Papanicolaou test cases accessioned between January 1, 2023, through March 31, 2025. The dataset included 512 517 cases accessioned with a total of 655 993 reviews (including CTs and pathologists). In total, 340 cases (*n* = 525 reviews) were excluded because no final diagnosis was available or there was no sign-out date at the time of collection. After exclusions, a total of 512 177 cases (*n* = 655 468 reviews) were included for analysis. The majority of cases (72.7%) were screened by a single reviewer (ie, CT alone, with no second CT or pathologist review).

Genius Dx implementation occurred on August 1, 2024. Cases from January 1, 2023, through June 30, 2024, were classified as pre–Genius Dx implementation; cases from August 1, 2024, through March 1, 2025, were classified as post–Genius Dx implementation.

Per-day caseload was calculated by tallying the number of cases with a diagnostic assessment provided based on review date. Reviewer ratio was calculated by dividing the number of cases reviewed per day by the number of reviewers, grouping CTs separately from pathologist totals. Sixty-two CTs reviewed cases during the 27-month time frame, with 422 506 reviews before Genius Dx implementation and 163 046 after Genius Dx implementation (an additional 2041 reviews occurred between April 1 and 7, 2025, on cases that were accessioned in the study period but were excluded from per-day caseload calculations because the full review total was incomplete for dates after March 31, 2025). Not all the CTs had case reviews before and after Genius Dx implementation, however. Per-day caseload estimates were calculated based on the number of reviewers overall as well as a focus on the 8 CTs who had consistent review volumes through March 2025, with 4 beginning in January 2024 and the remaining 4 beginning in June 2023. There were 20 pathologist reviewers across the 27-month time frame, with 42 943 reviews before Genius Dx implementation and 24 551 after (an additional 381 reviews occurred between April 1 and 7, 2025, but were excluded from per-day caseload calculations due to the March 31, 2025, cutoff).

Among the 140 328 cases with 2 or more reviewers, 65 060 were quality control cases (46.4% of the cases with ≥2 reviewers, 12.7% of overall cases). Timing from accession to review date and accession to sign-out date were included in the dataset.

Per day caseload, average review time, and average case times were compared using the independent-samples *t* test, with corresponding effect size (Cohen *d*) and 95% CIs for cases before vs after the August 1, 2024, Genius Dx implementation date. Pearson ꭓ^2^ test of independence with corresponding effect size (Cramer *V*) and adjusted standardized residual was used to determine whether there was a relationship between the proportion of cases per diagnostic category before and after Genius Dx implementation and quality control assessment in the 5 months before and after the initial 3-month period.

Statistical significance was set at *P* < .05. The magnitude of effect size of Cohen *d* was considered small (0.2-0.5), medium (0.5-0.8, or large (>0.8).^18^ The magnitude of effect size of Cramer *V* was considered negligible (0-0.05), weak (0.05-0.1), moderate (0.1-0.15), strong (0.15-0.25), or very strong (>0.25).^19^ Adjusted standardized residuals above 2.0 or below −2.0 are the general cutoff used to show greater discrepancy, for ɑ = .05. Analysis was performed using IBM SPSS Statistics version 30.0.0.0 (172) and Microsoft Excel for Microsoft 365, version 2503 (build 18623.20086).

## RESULTS

Table 1 shows the average overall per-day review totals by reviewer type (CT and pathologist), average number of reviewers, and ratio of cases per reviewer before and after Genius Dx implementation. The average number of CT reviews was statistically equivalent between time frames, with an average of 747.8 cases per day before Genius Dx and 758.4 cases per day after Genius Dx (*t*_321.6_ = −0.4; *P* = .69). There were statistically significantly fewer reviewers per day on average, however, after Genius Dx implementation (8.1 CTs per day) than before Genius Dx implementation (10.4 CTs per day) (*t*_501.2_ = 7.1, *P* < .001), with a medium effect size (Cohen *d* = 0.505). The ratio of cases per CT per day was statistically significantly higher after Genius Dx implementation (94.7 cases per day per CT) than before (74.5 cases per day per CT) (*t*_778.0_ = −14.1; *P* < .001), with a large effect size (Cohen *d* = −1.131).

**Table 1:** Average Number of Cases Reviewed, Number of Reviewers, and Ratio of Cases per Reviewer Before and After Genius Digital Diagnostics System Implementation

Per-day case summary			Mean	SD	Mean difference	*t*	*df*	*P* value	95% CI of mean difference	Cohen *d*
CT	Cases	Before^a^	747.8	274.2	−10.6	−0.4	321.6	.69	−62.2 to 41.1	−0.036
		After^b^	758.4	346.0						
	Reviewers	Before^a^	10.4	4.7	2.2	7.1	501.2	**<.001**	1.6-2.9	0.505
		After^b^	8.1	3.6						
	Ratio of cases per reviewer	Before^a^	74.5	16.7	−20.2	−14.1	778.0	**<.001**	−23.0 to −17.4	−1.131
		After^b^	94.7	20.6						
Pathologist	Reviews	Before^a^	106.0	46.4	−39.2	−7.5	251.7	**<.001**	−49.6 to −28.9	−0.766
		After^b^	145.3	61.4						
	Reviewers	Before^a^	4.4	1.3	1.0	8.0	572.0	**<.001**	0.7-1.2	0.732
		After^b^	3.4	1.4						
	Ratio of cases per reviewer	Before^a^	24.5	10.2	−22.8	−10.9	189.5	**<.001**	−26.9 to −18.6	−1.367
		After^b^	47.3	26.3						

a Before: January 1, 2023, to June 30, 2024 (*n* = 422 506 CT reviews, *n* = 42 943 Pathologist reviews).

b After: August 1, 2024, to March 31, 2025 (*n* = 163 046 CT reviews, *n* = 24 551 Pathologist reviews).

Bold *P* value signifies a statistically significant difference before and after implementation.

The average number of pathologist reviews per day was statistically significantly higher after Genius Dx implementation (145.3 cases per day) than before Genius Dx (106.0 cases per day) (*t*_251.7_ = −7.5; *P* < .001; medium effect size, Cohen *d* = −0.766), with fewer reviewers (3.4 reviewers per day after Genius Dx vs 4.4 reviewers per day before Genius Dx) (*t*_572.0_ = 8.0; *P* < .001; medium effect size, Cohen *d* = 0.732). The ratio of cases per pathologist per day was statistically significantly higher after Genius Dx implementation (47.3 cases per day per pathologist) than before (24.5 cases per day per pathologist) (*t*_189.5_ = −10.9; *P* < .001), with a large effect size (Cohen *d* = −1.367).

Table 2 shows the per-day case summary for the 8 CTs who consistently reviewed cases throughout the targeted time frame. Most of the CTs reviewed significantly more cases per day after Genius Dx implementation. Each of the 4 CTs who began in January 2024 statistically significantly reviewed more cases per day, with the largest difference by CT6, who increased from an average of 61.1 cases per day before Genius Dx to 122.5 cases per day after Genius Dx (*t*_295.2_ = −28.2; *P* < .001), with a large effect size (Cohen *d* = −3.180). Among the 4 CTs who started by June 2023, each reviewed more cases per day, with 3 of the 4 CTs having statistically significant differences before and after Genius Dx. The sole CT without a statistically significant increase was CT1, who increased from 94.3 cases per day to 98.9 cases per day, which was not statistically significantly different (*t*_240.1_ = −1.7; *P* = .090). The remaining 3 CTs had statistically significantly higher per-day caseloads after Genius Dx implementation, with the largest increase for CT2, who increased from 100.1 cases per day to 117.5 cases per day (*t*_209.0_ = −7.9; *P* < .001), with a large effect size (Cohen *d* = −0.919).

**Table 2:** Average Per-Day Case Summary for Each Reviewer Before and After Genius Digital Diagnostics System Implementation

Reviewer			Mean	SD	Mean difference	*t*	*df*	*P* value^a^	95% CI of mean difference	Cohen *d*
CT (June 2023-March 2025)	1	Before	94.3	20.8	−4.5	−1.7	240.1	.09	−9.8 to 0.7	−0.187
		After	98.9	29.8						
	2	Before	100.1	16.4	−17.4	−7.9	209.0	**<.001**	−21.7 to −13.0	−0.919
		After	117.5	23.1						
	3	Before	107.5	24.6	−11.4	−4.0	253.1	**<.001**	−16.9 to −5.8	−0.426
		After	118.9	30.4						
	4	Before	97.6	15.5	−10.1	−4.5	213.6	**<.001**	−14.5 to −5.7	−0.520
		After	107.7	25.0						
CT (January 2024-March 2025)	5	Before	49.1	12.7	−54.0	−27.4	270.3	**<.001**	−57.9 to −50.2	−3.025
		After	103.2	21.5						
	6	Before	61.1	16.4	−61.3	−28.2	295.2	**<.001**	−65.6 to −57.1	−3.180
		After	122.5	21.6						
	7	Before	68.1	13.3	−53.6	−30.7	287.1	**<.001**	−57.0 to −50.1	−3.522
		After	121.7	16.9						
	8	Before	44.5	9.4	−42.5	−24.9	245.9	**<.001**	−45.8 to −39.1	−2.724
		After	86.9	19.3						

a Bold *P* value signifies a statistically significant difference before and after implementation.

Table 3 shows the average time from accession to sign-out diagnosis before and after Genius Dx implementation by diagnostic category. Post–Genius Dx implementation screening time was statistically significantly shorter for diagnoses of negative; atypical squamous cells of uncertain significance (ASC-US); low-grade squamous intraepithelial lesion (LSIL); and atypical squamous cell, cannot exclude high-grade squamous intraepithelial lesion or higher (ASC-H+). Accession to sign-out time for cases signed out as negative was statistically significantly shorter after Genius Dx implementation (64.9 hours) than before (78.7 hours) (*t*_331794.8_ = 87.6; *P* < .001), with a small effect size (Cohen *d* = 0.245). Accession to sign-out time for cases signed out as ASC-US was statistically significantly shorter after Genius Dx implementation (88.5 hours) than before (104.1 hours) (*t*_12311.2_ = 18.3; *P* < .001), with a small effect size (Cohen *d* = 0.293). Accession to sign-out time for cases signed out as LSIL was statistically significantly shorter after Genius Dx implementation (86.0 hours) than before (102.4 hours) (*t*_7386.0_ = 13.3; *P* < .001), with a small effect size (Cohen *d* = 0.329). Accession to sign-out time for cases signed out as ASC-H+ was statistically significantly shorter after Genius Dx implementation (91.3 hours) than before (108.3 hours) (*t*_2688.0_ = 5.6; *P* < .001), with a small effect size (Cohen *d* = 0.233).

**Table 3:** Average Time in Hours From Accession to Sign-Out Diagnosis

Accession to sign-out			Mean, h	SD	Mean difference	*t*	*df*	*P* value^c^	95% CI of mean difference	Cohen *d*
Sign-out diagnosis	Unsatisfactory	Before^a^	97.9	62.7	−1.0	−0.8	6926.0	.42	−3.5 to 1.5	−0.018
		After^b^	98.9	46.7						
	Negative	Before^a^	78.7	60.5	13.8	87.6	331794.8	**<.001**	13.5-14.1	.245
		After^b^	64.9	43.5						
	ASC-US	Before^a^	104.1	53.6	15.6	18.3	12311.2	**<.001**	13.9-17.3	.293
		After^b^	88.5	52.7						
	LSIL	Before^a^	102.4	49.1	16.5	13.3	7386.0	**<.001**	14.0-18.9	.329
		After^b^	86.0	52.3						
	ASC-H+	Before^a^	108.3	80.4	17.0	5.6	2688.0	**<.001**	11.0-23.0	.233
		After^b^	91.3	52.4						

Abbreviations: ASC-H+, atypical squamous cell, cannot exclude high-grade squamous intraepithelial lesion; ASC-US, atypical squamous cells of uncertain significance; LSIL, low-grade squamous intraepithelial lesion.

a Before: January 1, 2023, to June 30, 2024 (*n* = 367 901).

b After: August 1, 2024, to March 31, 2025 (*n* = 144 279).

c Bold *P* value signifies a statistically significant difference before and after implementation.

When grouping the diagnostic categories based on clinical follow-up (unsatisfactory and negative, ASC-US+), the average time from accession to sign-out for cases signed out as unsatisfactory or negative was 78.6 hours before Genius Dx compared with 64.9 hours after Genius Dx (*t*_335787.9_ = 88.4; *P* < .001), with a small effect size (Cohen *d* = 0.246)). The average time from accession to sign-out for cases signed out as ASC-US+ was 103.3 hours before Genius Dx compared with 87.1 hours after Genius Dx (*t*_38629_ = 23.4; *P* < .001), with a small effect size (Cohen *d* = 0.248)). Table 4 shows the distribution of sign-out diagnosis before and after Genius Dx implementation.

**Table 4:** Proportion of Case Sign-Out Diagnostic Categories Before and After Genius Dx Implementation

		Before Genius Dx			After Genius Dx			χ^2^	*df*	*P* value^a^	Cramer *V*
		No.	%	Adjusted standard residual	No.	%	Adjusted standard residual				
Sign-out diagnosis	Unsatisfactory	4918	1.3	−14.1	2691	1.9	14.1	704.35	4	**<.001**	0.037
	Negative	345 038	93.8	26.0	132 385	91.8	−26.0				
	ASC-US	11 120	3.0	−19.7	5947	4.1	19.7				
	LSIL	4964	1.3	−8.9	2424	1.7	8.9				
	ASC-H+	1861	0.5	−3.1	829	0.6	3.1				

Abbreviations: ASC-H+, atypical squamous cell, cannot exclude high-grade squamous intraepithelial lesion; ASC-US, atypical squamous cells of uncertain significance; Genius Dx, Genius Digital Diagnostics System; LSIL, low-grade squamous intraepithelial lesion.

a Bold P value signifies a statistically significant difference Before and After implementation

The proportion of diagnostic categories statistically significantly differed by Genius Dx implementation, particularly with fewer negative cases after Genius Dx implementation (91.8%) than before (93.8%) when looking at the largest adjusted standard residuals ($\chi_{4}^{2}$ = 704.35; *P* < .001), but the effect size was negligible (Cramer *V* = 0.037). In addition, the largest gap in diagnostic categories is the 2.0-percentage-point difference in negative cases, which is not a clinically meaningful difference (Table 5).

**Table 5:** Difference in Proportion of Diagnostic Categories Before and After Genius Dx Implementation

	Sign-out diagnosis, %		
	Before	After	Difference (before ‒ after)
Unsatisfactory	1.3	1.9	0.5
Negative	93.8	91.8	−2.0
ASC-US	3.0	4.1	1.1
LSIL	1.3	1.7	0.3
ASC-H+	0.5	0.6	0.1

Abbreviations: ASC-H+, atypical squamous cell, cannot exclude high-grade squamous intraepithelial lesion; ASC-US, atypical squamous cells of uncertain significance; Genius Dx, Genius Digital Diagnostics System; LSIL, low-grade squamous intraepithelial lesion.

## DISCUSSION

Cytology is an integral part of cervical cancer screening in the United States. Despite its importance, cytology laboratories are facing substantial workforce shortages, particularly with respect to CTs. This shortage is critically important given the labor-intensive nature of cervical cytology review. The Genius Dx has been Food and Drug Administration cleared since February 2024, with many US laboratories adopting it for routine cervical cancer screening. Although several studies have demonstrated that review time and glass hands-on time is decreased when using the Genius Dx, these studies were done in an experimental setting.^13^^,^^17^ To date, we believe that our study is the first in the United States to use real-world data before and after Genius Dx implementation in routine clinical practice to demonstrate substantial improvements in workflow efficiency in a high-volume cytology laboratory.

In the first 8 months after Genius Dx adoption, the turnaround time from accessioning to sign-out of the case was statistically significantly reduced for all major diagnostic categories, despite no significant difference in case volume and with fewer CTs and pathologists reviewing cases. The greatest reduction in turnaround time was observed in the most severe disease cases (ASC-H+). This finding highlights the value of the reduction in glass slide movement and review time for CTs and pathologists. These results also demonstrate the patient benefit of improving the turnaround time most in cases with the highest grade of disease.

There was no difference in the daily number of cases being screened before and after Genius Dx implementation. After implementation, fewer CTs and pathologists were needed to screen the same number of daily cases, demonstrating that Genius Dx enables greater efficiency, potentially mitigating the effects of workforce shortages and expanding responsibilities that have challenged cytology laboratories in recent years.^7^^,^^9^

A more in-depth analysis of the 8 CTs who were consistently screening cases before and after Genius Dx implementation found that CTs screened, on average, 53.5% more cases in the 8 months after Genius Dx implementation. The increase in efficiency varied across the 8 CTs, with a greater increase in efficiency observed in the second 4 CTs than in the first 4. This shift could be due to the first 4 CTs not being solely responsible for screening cervical cytology cases and completing many other tasks in the laboratory each day. The main task that could have affected any efficiency gains from adoption of the Genius Dx is quality control rescreening. This group of CTs performs all our laboratory’s quality control reviews, which is done in our laboratory as a full manual review of the glass slide using a light microscope. These CTs also perform tasks such as 5-year look-backs and nongynecologic cytology screening, which are also done on a light microscope manually. In addition, this group of 4 CTs were more experienced and already screening at a relatively high rate before implementation of Genius Dx. In our analysis, we had access to the overall number of cases screened per day by each CTs and not the breakdown of how those cases were screened—whether they were screened using the Genius Dx or manually using the microscope. Nevertheless, all but one of these CTs substantially increased the number of cases reviewed each day.

The second 4 CTs (5-8) were newer cytologists and were dedicated to screening only cervical cytology cases using Genius Dx. Each of these CTs screened a statistically significantly higher number of cases per day after transitioning to Genius Dx. Given that these CTs were junior, it is possible that some of the efficiency gains observed in this study could have been realized with additional experience with their previous screening method (ThinPrep Imaging System). The results from this study, however, are consistent with those from our previous experimental study, which demonstrated similar increases in efficiency using the Genius Dx compared with the ThinPrep Imaging system when the CTs were dedicated to screening and did not have additional tasks.^17^ In that study, the 2 CTs were senior cytologists who are not typically dedicated to screening Papanicolaou tests.

Given the workforce shortages, it was important to demonstrate improvements in efficiency when screening cervical cytology with Genius Dx. It is also critical to ensure that these improvements were not at the expense of diagnostic accuracy. In this study, there was a small but statistically significant decrease in the diagnosis of negative cytology and an increase in detection across all disease categories (ASC-US, LSIL, ASC-H+) after Genius Dx implementation. These data confirm that the statistically significant increases in efficiency observed were also associated with increased disease detection in our laboratory after implementation of Genius Dx. Our study showed similar increases in disease detection to a study published by Doxtader et al,^13^ who completed a retrospective study with biopsy-confirmed disease and demonstrated increased detection of severe disease compared with microscope review. Our study differed in that we did not correlate to biopsy data for these cases to confirm these increases in disease detection. It is possible that some of the increase in disease detection is due to learning curve rather than to true increase disease detection.

Although these data confirm the value of Genius Dx in real-world practice through a retrospective design using the LIS, certain limitations must be acknowledged. The study was conducted within a single large laboratory and relied on information stored within the LIS to determine turnaround time, CT and pathologist workload, and distribution of cases within diagnostic categories. Many factors can skew laboratory data, such as time of year, changes in staff, changes in job functions, vacations, and illnesses. All these factors could potentially affect the interpretation of results pulled from the LIS. This study benefits from a large dataset of more than half a million Papanicolaou test results and the fact that CTs are required to track their daily screening numbers as part of 1988 Clinical Laboratory Improvement Amendments. Nevertheless, every laboratory has differences in workflow practices and case complexity that may result in different findings. In addition, although acclimatization periods were considered, longer-term impacts on efficiency and diagnostic concordance warrant further investigation. Future studies should confirm whether similar results are observed in other settings and other time periods to make a broader assessment about workforce implications.

The real-world implementation of the Genius Digital Diagnostics System in a high-volume cytology laboratory resulted in substantially increased reviewer efficiency and reduced turnaround times. Genius Dx addresses key workflow challenges facing modern cytology laboratories and supports greater case throughput per cytologist and pathologist without compromising disease diagnoses. These findings provide compelling evidence for the adoption of digital cytology platforms to address workforce challenges by enhancing laboratory efficiency.

## Data Availability

The data that support the findings of this study are available from the corresponding author upon reasonable request.
